# Migratory Sleeplessness in the White-Crowned Sparrow *(Zonotrichia leucophrys gambelii)*

**DOI:** 10.1371/journal.pbio.0020212

**Published:** 2004-07-13

**Authors:** Niels C Rattenborg, Bruce H Mandt, William H Obermeyer, Peter J Winsauer, Reto Huber, Martin Wikelski, Ruth M Benca

**Affiliations:** **1**Department of Psychiatry, University of WisconsinMadison, Wisconsin, United States of America; **2**Department of Pharmacology and Experimental Therapeutics, Louisiana State UniversityNew Orleans, Louisiana, United States of America; **3**Department of Ecology and Evolutionary Biology, Princeton UniversityPrinceton, New JerseyUnited States of America

## Abstract

Twice a year, normally diurnal songbirds engage in long-distance nocturnal migrations between their wintering and breeding grounds. If and how songbirds sleep during these periods of increased activity has remained a mystery. We used a combination of electrophysiological recording and neurobehavioral testing to characterize seasonal changes in sleep and cognition in captive white-crowned sparrows *(Zonotrichia leucophrys gambelii)* across nonmigratory and migratory seasons. Compared to sparrows in a nonmigratory state, migratory sparrows spent approximately two-thirds less time sleeping. Despite reducing sleep during migration, accuracy and responding on a repeated-acquisition task remained at a high level in sparrows in a migratory state. This resistance to sleep loss during the prolonged migratory season is in direct contrast to the decline in accuracy and responding observed following as little as one night of experimenter-induced sleep restriction in the same birds during the nonmigratory season. Our results suggest that despite being adversely affected by sleep loss during the nonmigratory season, songbirds exhibit an unprecedented capacity to reduce sleep during migration for long periods of time without associated deficits in cognitive function. Understanding the mechanisms that mediate migratory sleeplessness may provide insights into the etiology of changes in sleep and behavior in seasonal mood disorders, as well as into the functions of sleep itself.

## Abstract 2

Neurophysiological and behavioural studies suggest that sleep- loss during the migratory season does not adversely affect cognitive capacity in captive white-crowned sparrows

## Introduction

Seasonal behaviors occur in virtually all organisms, ranging from insects to mammals (Goldman et al. 2004). Just as circadian rhythms allow organisms to anticipate daily cycles of light and dark, circannual behaviors such as migration allow them to predict and respond to seasonal changes in environmental conditions. Like circadian rhythms, seasonal migratory behavior is both endogenously generated and shaped by external factors such as photoperiod length, weather, and food availability (Gwinner and Helm 2003).

The most extraordinary examples of seasonal migration occur in birds, many species of which regularly migrate thousands of kilometers. Given the long distances traversed during migration, much research has focused on the timing of migratory flights, navigation during migration, and the energetic costs of migration (Berthold 1996; Gauthreaux 1996). One aspect of migration that is likely to impact all others, however, remains a complete mystery: Do birds sleep during migration and if so, how (Moore 1999; Schwilch et al. 2002; Jenni and Schaub 2003)?

In many bird species, migration proceeds at a pace that does not seem to allow much time for sleep. The apparent conflict between migratory behavior and sleep may be particularly extreme for songbirds. In the nonmigratory seasons, songbirds sleep at night and are active during the day. In the migratory seasons, however, many songbirds undergo a profound behavioral shift and begin to fly at night while still remaining active during the day (Berthold 1996). In the only study to directly observe migratory behavior using telemetry, a Swainson's thrush *(Catharus ustulatus)* flew on six of seven nights and traveled 1,500 km, with flights that occurred under favorable weather conditions lasting up to7 h (Cochran 1987; see also, Cochran and Wikelski 2004; Cochran et al. 2004); however, daytime activity was not reported. Although some studies have observed brief periods of sleep behavior in the evening prior to the initiation of a nocturnal flight (Eyster 1954; Berthold and Querner 1988; Berthold 1996; Ramenofsky et al. 2003), the overall increase in activity during migration suggests a marked reduction in time available for sleep.

Despite their apparent sleep loss, migrating songbirds are capable of engaging in adaptive waking behaviors, including prolonged flight, navigation, foraging, and evading predators in novel environments. The preservation of cognitive and physical performance during migration is surprising because sleep restriction in other animals causes profound deficits in neurobehavioral and physiological function. In humans, as little as one night of sleep deprivation adversely affects alertness, working memory, cognitive throughput (Van Dongen et al. 2003), divergent thinking (Wimmer et al. 1992; Harrison and Horne 1999), insight (Wagner et al. 2004), and memory consolidation (Karni et al. 1994; Stickgold et al. 2000, 2001; Maquet 2001; Fischer et al. 2002; Fenn et al. 2003), but see Siegel (2001). Even partial sleep restriction can have adverse effects on neurobehavioral function in humans; limiting sleep to 6 h per night (75% of the normal requirement) for ten nights decreases alertness to a level comparable to that following 24 h of total sleep deprivation (Van Dongen et al. 2003). The most prolonged sleep deprivation studies have been performed in rats, where near-total (>90%) sleep deprivation leads to physiological impairment culminating in death after as little as 2–3 wk (Rechtschaffen et al. 1983; Rechtschaffen and Bergmann 2002). Similarly, fruit flies *(Drosophila melanogaster)* deprived of sleep also die, suggesting that sleep serves a function vital to survival in all animals (Shaw et al. 2002).

Given the dramatic effects of sleep deprivation in other animals, the preservation of adaptive waking function in spite of the apparent reduction in sleep during migration in songbirds seems paradoxical. Songbirds might have found a way to obtain sufficient amounts of sleep by either engaging in short but intense bouts of sleep or by sleeping in flight. Alternatively, songbirds might possess a capacity unprecedented in the animal kingdom to circumvent or withstand the effects of sleep loss during migration.

We have performed long-term electrophysiological recordings of sleep and wakefulness during the nonmigratory and migratory seasons in a songbird, the white-crowned sparrow *(Zonotrichia leucophrys gambelii),* which migrates 4,300 km twice a year between Alaska and southern California (DeWolfe 1968; Chilton et al. 1995). We chose to study sleep in the white-crowned sparrow since migration has been investigated for over 50 years in this species, both in the wild and in captivity (Farner 1950; Ramenofsky et al. 2003). Furthermore, we measured cognitive function across an entire year to determine whether sleep loss has a differential effect on cognitive function during migratory and nonmigratory seasons.

## Results

### Demonstration of Migratory Restlessness in the Laboratory Setting

The sleep patterns of birds in a migratory state were recorded in captivity, where migratory behavior manifests itself as migratory restlessness *(Zugunruhe),* i.e., nocturnal activity, including hopping and wing flapping (Berthold and Querner 1988; Berthold et al. 2000). Migratory restlessness is genetically controlled (Berthold and Querner 1981; Berthold 1990) and appears to reflect the natural migratory urge, since the number of nights during which birds exhibit episodes of migratory restlessness is positively correlated with the duration of migration in the wild (Gwinner 1986; Berthold and Querner 1988; Berthold 1996).

**Figure 1:**
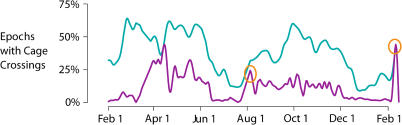
Activity Across the Year The purple line traces the (smoothed) percentage of 30-s epochs at night during which a bird (no. 38) broke an infrared beam by crossing the cage. The blue line shows the same information for the hours of light. Note the broad peak in activity between March and June and the broader and lower peak in activity between August and December, corresponding to the spring and fall migrations, respectively. The low levels of activity during July and December through March correspond to the summer and winter nonmigratory periods. The sharp high peaks (orange ovals) in early August of 2003 and early February of 2004 mark brief periods of experimenter-induced sleep restriction.

Starting in June 2002, we established colonies of white-crowned sparrows at the University of Wisconsin-Madison. Seasonal changes in activity were continuously recorded with an infrared activity monitoring system. As shown in a representative activity plot spanning one calendar year (Figure 1) activity in the dark phase, as well as in the light phase, increased dramatically during the migratory seasons in the spring (March–June) and fall (August–December), when these birds would normally be migrating between California and Alaska (Chilton et al. 1995). As indicated in previous reports using similar techniques, the increase in activity was more pronounced in the spring than in the fall (Berthold 1996). The activity data demonstrate that the typical patterns of seasonal changes in migratory restlessness could be reproduced reliably in our laboratory setting.

To further characterize seasonal changes in behavior, we used infrared-sensitive cameras to record the sparrows' behavior. As reported previously (Berthold and Querner 1988; Berthold et al. 2000), the video recordings revealed that the infrared activity monitoring system underestimated the amount of time that birds were active. In addition to hopping back and forth across the infrared beam, the birds also spent a significant amount of time either hopping over or under the infrared beam. Occasionally, birds also flapped their wings with their heads raised while holding onto a perch, as if attempting to initiate flight (Video 1) a behavior that was restricted to the night and virtually never occurred in nonmigratory birds. The video recordings thus confirmed that the birds were displaying migratory behaviors consistent with previous reports in songbirds (Berthold and Querner 1988; Berthold et al. 2000), including white-crowned sparrows (Ramenofsky et al. 2003), and indicated that they were spending even more time active and behaviorally awake during migration than was indicated by the infrared activity monitoring system.

### Electrophysiological Correlates of Sleep and Wakefulness in Sparrows

Although infrared activity and video monitoring suggested a reduction in the amount of sleep during migration, electrophysiological recordings are required to confirm behavioral state, as well as quantify potential changes in sleep stages and intensity. To characterize sleep patterns during the nonmigratory and migratory seasons, eight sparrows were instrumented for recording electroencephalographic activity (EEG) from both hemispheres, as well as electromyographic activity (EMG) (see Materials and Methods). To control for potential seasonal changes in sleep patterns related to the acute effects of photoperiod, all recordings were performed under a constant 12:12 light/dark (LD) cycle. Consequently, the seasonal changes in sleep patterns reported below can be attributed to endogenous changes in migratory state, rather than to the combined effects of migratory state and changes in photoperiod on behavior.

All of the birds instrumented for EEG and EMG recording exhibited episodes of migratory restlessness starting between mid-July and mid-August, in synchrony with the other birds in the laboratory. Their behavior was indistinguishable from that displayed by the sparrows without electrophysiological implants. In three birds, bouts of migratory restlessness started prior to surgery or during postoperative recovery.

The electrophysiological correlates of sleep and wakefulness in nonmigrating and migrating white-crowned sparrows were similar to each other, as well as to those previously described in other species of birds (Rattenborg and Amlaner 2002).

#### Wakefulness

**Figure 2:**
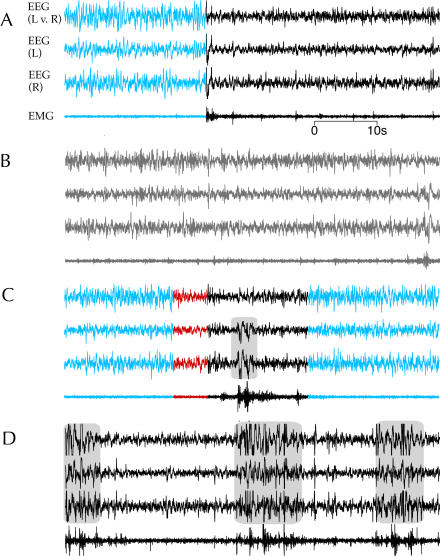
Examples of Electrophysiological Correlates of Behavioral States This figure shows four 1-min samples each containing three EEG recordings (left hemisphere vs. right hemisphere [L v. R], left hemisphere vs. neutral reference [L], and right hemisphere vs. neutral reference [R]) and one EMG from one sparrow (no. 65) depicting the typical electrophysiological correlates of each behavioral state. (A) Transition from SWS (blue) to wakefulness (black). (B) Drowsiness (gray). (C) Transition from SWS (blue) to a bout of REM sleep (red), and then a brief awakening (black), followed by a return to SWS (blue). (D) Wakefulness during a period of migratory restlessness. High-amplitude artifacts associated with gross movements are shaded with a gray background. (A–C) were recorded on 11 August 2003, during the summer while the bird was in a nonmigratory state, and (D) was recorded on 11 October 2003, during the fall migratory period.

Behavior during wakefulness included hopping and flying around the cage, feeding, drinking, feather preening, and actively scanning the room. Although movement artifacts obscured the EEG and EMG during gross movements (e.g., hopping and wing flapping), the recordings occurring immediately before and after such behaviors showed a low-amplitude, activated EEG in both hemispheres, and high EMG activity typical of alert wakefulness (Figure 2).

#### Drowsiness

Upon ceasing active wakeful behaviors, the sparrows entered drowsiness, a mixed state with behavioral and EEG characteristics of both wakefulness and slow-wave sleep (SWS) (Campbell and Tobler 1984). Sparrows in this state typically stood on the floor facing the front of the cages with their heads facing toward the center of the room (Video 2). Their heads were held close to their bodies, and the position of the eyelids fluctuated between open, partially closed, and completely closed states, while their heads moved from side to side. This behavior was intermittently interrupted by the birds opening their eyes completely and raising their heads, apparently attending to the activities of other birds in the room. The fact that the sparrows selected optimal vantage points in their cages from which to monitor the room, moved their heads from side to side in a scanning manner, and intermittently responded to stimuli in the environment indicates that several behavioral aspects of wakefulness were intact during this state. Although artifact from the head movements precluded reliable spectral analysis of the EEG during drowsiness, as in other studies of songbirds (Szymczak et al. 1993), visual assessments of the EEG showed activity intermediate between that of wakefulness and SWS (i.e., increased amplitude in the low-frequency range relative to wakefulness; Figure 2B). The EMG typically showed brief bursts of activity associated with head movements during drowsiness. The EEG amplitude and frequency approached that of SWS, but unlike unequivocal SWS, which is followed by rapid eye movement (REM) sleep in birds and mammals (Amlaner and Ball 1994; Zepelin 2000), REM sleep never occurred directly following drowsiness. Based on the mixture of behavioral and electrophysiological features of wakefulness and SWS, drowsiness was not included in either the calculation of time in wakefulness or sleep, but rather it constituted a separate behavioral category.

#### Slow-wave sleep

In contrast to the vigilant behaviors exhibited by sparrows in drowsiness, during SWS the birds were motionless, usually with closed eyes; the head was either pulled in toward the body and facing forward or resting on the bird's back (Video 3). The amplitude of low-frequency EEG reached its highest levels during SWS (see Figure 2A and 2C). As in other birds (Spooner 1964; Ookawa and Gotoh 1965; Ball et al. 1988; Rattenborg et al. 1999b), sparrows occasionally showed interhemispheric asymmetries in EEG low-frequency activity during SWS. However, such asymmetries were restricted to periods of immobility, and they never occurred during active behaviors.

#### REM sleep

An activated EEG pattern similar to that occurring during wakefulness characterized REM sleep (Figure 2C). Unlike most mammals, but similar to other birds, EMG activity recorded from the nuchal muscles rarely declined during REM sleep. Nevertheless, behavioral signs of reduced muscle tone made REM sleep readily distinguishable from brief awakenings; in contrast to wakefulness, during REM sleep the eyes were closed and the head either rolled to one side or fell forward, occasionally dropping to the bird's feet. In extreme cases, birds would sway and briefly lose their balance on the perch. Episodes of REM sleep typically lasted a maximum of 10 s, but often occurred in clusters separated by only a few seconds of SWS.

#### Migratory restlessness

During episodes of migratory restlessness, defined as active hopping and wing flapping at night interrupted by only brief pauses in motor activity, both eyes were completely open, and the EEG recorded from both hemispheres showed an activated pattern, indistinguishable from alert wakefulness (Figure 2D). There was no sign of either drowsiness or an interhemispheric asymmetry in SWS-related EEG, indicating that sparrows exhibiting migratory restlessness were completely awake.

### Comparisons of Sleep Patterns in Nonmigrating and Migrating Sparrows

**Figure 3:**
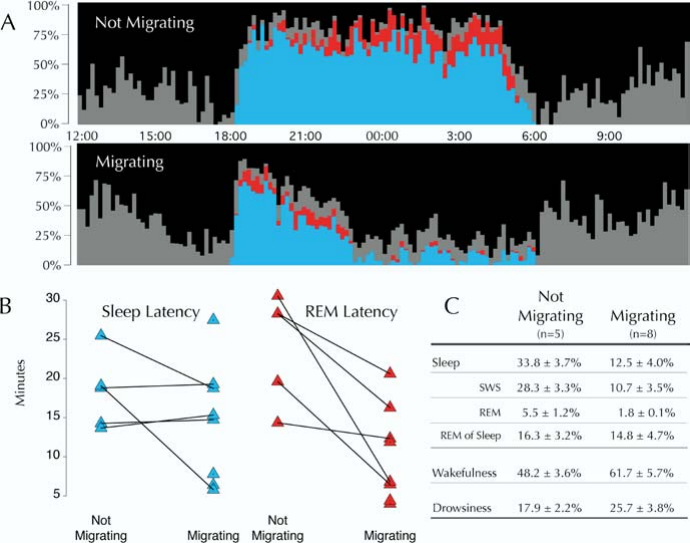
Changes in Sleep during Fall Migration Behavioral state was scored across 24-h (noon to noon) periods using a combination of video and electrophysiological recordings for birds in a nonmigratory (*n *= 5) and migratory (*n* = 8) state. The plots and table reflect the average for all birds in each group. All recordings were performed under a 12:12 LD photoperiod with lights turned off at 18:00 and on at 06:00. (A) Proportion of time in each behavioral state for nonmigrating (top) and migrating (bottom) birds. The proportion of every 10-min period spent in each sleep/wakefulness state was calculated for each bird and then averaged across all birds: wakefulness (black), drowsiness (gray), SWS (blue), and REM sleep (red). Note that overall sleep propensity in migrating birds is greatly diminished between approximately 22:30 and 06:00. Note also the increased propensity for REM sleep from 18:00 to 20:00 as compared to the same time period when not migrating. (B) Sleep and REM sleep latencies. Sleep latency was calculated as the length of time from lights out until the first occurrence of sleep (in all cases SWS) for birds in a nonmigratory and migratory state; average sleep latency did not differ significantly between nonmigrating and migrating birds. REM sleep latency was calculated as the length of time from sleep onset to the first occurrence of REM sleep. Note that REM sleep occurred earlier in sleep during migration for all five birds that were recorded in both a nonmigratory and migratory state (*t* = 3.3, paired, two-tailed, *p* < 0.05). Note also that the REM sleep latencies for the three birds recorded only in a migratory state were shorter than the shortest REM sleep latency in nonmigrating birds. (C) Sleep percentages. Average daily percentages of sleep and wakefulness states for birds in a nonmigrating (*n* = 5) and migrating (*n* = 8) state. Total sleep is the sum of SWS and REM sleep. For all states of vigilance, values for the migrating condition differed significantly from the nonmigrating condition (*p* < 0.01, after Bonferroni correction). The proportion of total sleep occupied by REM sleep was not significantly different between migratory states.

In nonmigrating sparrows, sleep and wakefulness patterns across the day were typical of those previously described in a songbird (Szymczak et al. 1993). The proportion of time spent in each state varied in a consistent manner across the LD cycle (Figure 3A). Wakefulness and drowsiness encompassed all of the time during the light phase and a small proportion of time during the dark phase. At night, SWS was the predominant sleep stage during all hours of the night. In a pattern similar to humans and other mammals (Borbély and Achermann 2000; Tobler 2000), the proportion of time spent in SWS declined across the night, whereas REM sleep increased.

The migratory state was marked by a dramatic change in both the amount and pattern of sleep across the day. The most striking difference was a large reduction in total amount of sleep in migrating birds. Figure 3A shows the average hypnogram for all birds recorded during nonmigratory (*n* = 5) and migratory (*n* = 8) conditions. Total time spent sleeping was reduced by an average of 63% in migrating birds compared to nonmigrating birds (Figure 3C). In the most extreme case, sleep time decreased from 9.05 h on a nonmigrating night to 1.39 h on a migrating night, representing a sleep reduction of about 85%. In all but one bird, most of the sleep obtained on migratory nights occurred during the first few hours of the night. One bird, however, obtained more sleep during the second half of the night, although it had a brief episode of sleep at the beginning of the night, followed by episodes of migratory restlessness.

**Figure 4:**
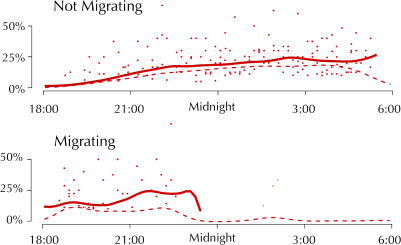
REM Sleep across the Dark Phase REM sleep as a proportion of total sleep time is plotted for every 10-min period during the dark phase for birds in nonmigrating (*n* = 5) and migrating (*n* = 8) states. The individual dots represent the average for each 10-min period; the solid line is a spline fit to these data. The dashed line represents the absolute amount of REM sleep, as a percentage of recording time. Note that the fit for the migrating birds is truncated, not only because very few periods after midnight had any REM sleep, but also because no point after midnight was based on more than one bird.

In addition to the restriction of sleep to the first few hours of the night in most migrating birds, there was also a shift in the timing of REM sleep. Although SWS latency was not affected by migratory status, the latency from SWS onset to REM sleep onset changed from 24.2 ± 6.9 min on nonmigratory nights to 10.3 ± 5.9 min on migratory nights (Figure 3B). Even in the bird that slept more during the second half of the night, REM sleep still occurred unusually early during the brief initial bout of sleep on its migratory night. In addition, REM sleep latencies for the three birds recorded only in a migratory state were all shorter than the shortest REM sleep latency for the five birds recorded in a nonmigratory state. Moreover, REM sleep as a proportion of total sleep time was elevated early in migratory nights (i.e., 18:00–20:00) when compared to the corresponding hours on nonmigrating nights (10.0% vs. 2.8%, *t* = 2.5, *p* < 0.05) (Figure 4). Although the migratory state significantly influenced the timing of REM sleep, the overall proportion of total sleep time spent in REM sleep was similar on nonmigratory (16.3%) and migratory (14.8%) nights (*t* = 0.56, *p* > 0.1) (see Figure 3C).

Finally, despite the marked reduction in nocturnal sleep during migration, sleep did not occur during the light phase in migrating birds; as in nonmigrating birds, SWS and REM sleep were restricted to the dark phase of the LD cycle. Nonetheless, time spent in drowsiness increased significantly during the light phase (38.4% vs. 27.7%, *t* = 2.68, *p* < 0.05) in migrating birds; drowsiness also increased during the dark phase, but this did not reach statistical significance (12.9% vs. 8.1%, *t* = 1.73, *p* > 0.1). On migratory nights, drowsiness usually occurred during the later half of night between bouts of migratory restlessness.

### Spectral Analysis of the SWS EEG in Nonmigrating and Migrating Sparrows

**Figure 5:**
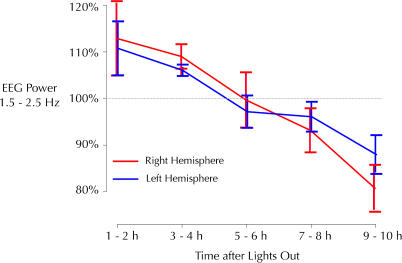
Time Course of EEG Power Density in the 1.5- to 2.5-Hz Band in SWS This figure shows the time course of EEG power density in the 1.5- to 2.5-Hz band in SWS during the dark phase for the left (blue) and right (red) hemisphere in nonmigrating sparrows. Curves represent mean 2-h values with standard error of the mean (*n* = 5). The EEG power density in the 1.5- to 2.5-Hz band of each 2-h interval is expressed as a percentage of the average EEG power in the 1.5- to 2.5-Hz band over all SWS epochs (dashed line = 100% of average 1.5- to 2.5-Hz power). The last 2-h interval is excluded since not all birds exhibited SWS during this time. A two-way, repeated measures ANOVA, with “hemisphere” and “2-h intervals” as factors, revealed a significant effect of the 2-h interval (*F* = 5.60, *p* < 0.05 with the Greenhouse–Geisser correction); neither an effect of hemisphere, nor an interaction between hemisphere and interval reached statistical significance (*p* > 0.1).

In addition to determining changes in the amount and type of sleep, we also compared EEG activity during SWS on nonmigratory and migratory nights for evidence of changes in sleep intensity using fast Fourier transform (FFT) spectral analysis of the EEG. In mammals, SWS-related slow-wave activity (SWA) of 0.75- to 4.5-Hz appears to reflect sleep intensity, since arousal thresholds are positively correlated with the amount of SWA (Franken et al. 1991; Neckelmann and Ursin 1993). SWA in the 0.75- to 4.5-Hz band also increases as a function of prior time awake and shows a progressive decline across the sleep period in mammals, suggesting that SWA is an EEG marker of sleep-related homeostatic processes (Borbély 1982; Tobler 2000). We first examined the time course of SWA across the night on nonmigratory nights using the 0.75- to 4.5-Hz frequency band typically employed in mammals, but we were unable to detect a significant decline in spectral power. When we examined changes in spectral power across all frequencies, however, a significant and pronounced decline across the night was apparent in the 1.25- to 2.5-Hz band for both the left and right hemispheres (Figure 5). Assuming that EEG in this frequency range reflects homeostatic processes in sparrows, and given the marked reduction in sleep during migration, we predicted that sparrows might compensate for decrements in total amounts of sleep with increased spectral power in the 1.25- to 2.5-Hz range during SWS on migratory nights. In the five birds recorded on both nonmigratory and migratory nights, however, we did not detect a significant increase in 1.25- to 2.5-Hz spectral power during SWS on the migratory night when compared to the corresponding hours of the nonmigratory night; three birds showed an increase and two birds showed a decrease in 1.25- to 2.5-Hz spectral power.

### Assessing Cognitive Function in Sparrows

Given the reduction in time spent sleeping and the apparent lack of a compensatory increase in sleep intensity during migration, we were interested in determining whether sparrows in a migratory state showed associated changes in cognitive function. Because we were interested in detecting seasonal changes in cognition, we used a test that could be administered repeatedly to the sparrows over long periods of time and without the potential confound of “practice” effects, a repeated-acquisition task; it has been widely used in humans and animals to provide repeated measures of the acute and chronic effects of neurotoxic insult on the ability to acquire a new sequence of operant responses (Winsauer et al. 2002). When combined with a performance component that simply requires memory of a previously learned sequence of operant responses, these two tasks can be used to determine whether changes in responding and accuracy during acquisition are related to direct effects on learning or global effects on psychomotor performance. The repeated-acquisition procedure can also be used to assess the effects of sleep deprivation on both the quality (i.e., accuracy) and quantity (i.e., number of responses) of behavior (Cohn et al. 1992).

We trained a group of eight sparrows not instrumented for electrophysiological recordings to respond under a multiple schedule of repeated-acquisition and performance in standard operant testing chambers (see Materials and Methods). In the acquisition component, birds learned a different three-response sequence of key pecks (e.g., left-right-center) during each session under a second-order fixed-ratio (FR) 3 schedule. In contrast, during the performance component, birds responded on the same three-response sequence each session under the same schedule of reinforcement. Despite being wild-caught, the sparrows adapted well to the testing apparatus and readily learned to respond in both tasks (Video 4).

### Effects of Sleep Restriction on Cognitive Function during the Nonmigratory Season

**Figure 6:**
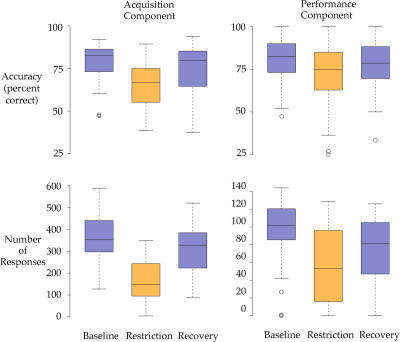
Operant Behavior and Sleep Restriction Average values for accuracy and the total number of responses on the acquisition and performance tasks are shown for the 3 d preceding sleep restriction, the 3 d of sleep restriction, and the 3 d following sleep restriction; boxes represent the 25th to 75th percentile of data with the median indicated by the line across the box. The “whiskers” extend from the quartiles to the most extreme value less than 1.5 times the interquartile range. Points outside the whiskers are plotted with small circles. Accuracy and total number of responses decreased significantly in both the acquisition and performance tasks following sleep restriction (*n* = 7).

To determine whether the task was sensitive to the effects of sleep restriction, as well as to provide a comparison for the effects of sleep loss occurring spontaneously during migration, sleep was restricted to the first 3 h of the dark phase on three consecutive nights during the nonmigratory (winter) season (see Materials and Methods). A 3-h sleep period at the start of the dark phase was chosen to mimic the general sleep pattern of sparrows during migration. Sleep restriction reduced accuracy (percentage correct responses) on both the acquisition (repeated measures analysis of variance [ANOVA], F = 12.65, p < 0.001) and performance (F = 3.12, p < 0.05) components of the task (Figure 6). The total number of responses also decreased following sleep restriction in both the acquisition (F = 33.91, p < 0.0001) and performance (F = 10.25, p < 0.0001) components of the task (see Figure 6). These effects of sleep restriction were evident following the first night and persisted following subsequent nights of sleep restriction.

### Comparison of Cognitive Function during Migratory and Nonmigratory Seasons

**Figure 7:**
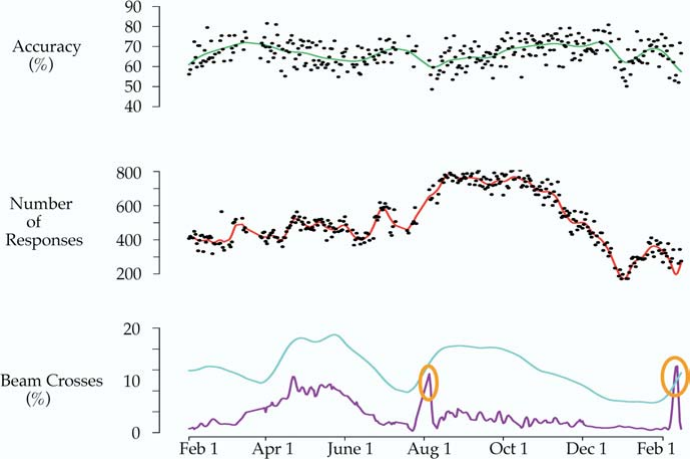
Comparison of Operant Responding and Migratory Behavior The average value for all birds of nighttime (purple) and daytime (blue) activity (percentage of 30-s epochs containing at least one infrared beam break) and the accuracy (green) and total number of responses (red) on the acquisition task (*n* = 8). As in Figure 1, the sharp high peaks (orange ovals) in early August of 2003 and early February of 2004 mark brief periods of experimenter-induced sleep restriction.

The same group of eight sparrows was tested under the multiple schedule of repeated-acquisition and performance for one year, encompassing both spring and fall migrations. Figure 7 shows accuracy, the total number of responses in the repeated-acquisition component of the task, and nocturnal activity across the year for the entire group of birds. During periods of increased nocturnal activity corresponding to the spring (March–June) and fall (August–December) migratory periods, accuracy on the repeated-acquisition task remained stable. The number of responses was lowest during the winter, intermediate during the spring migration and summer, and actually reached the highest level during the fall migration, before returning to the low winter levels.

**Figure 8:**
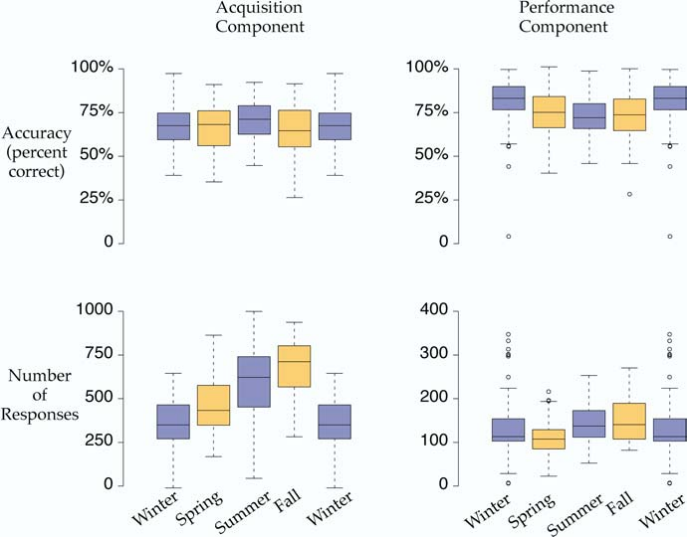
Seasonal Aspects of Operant Behavior Accuracy and the total number of responses during the acquisition task are shown for the 3 wk of spring and fall during which each bird was most active (orange) at night and for the 3 wk during summer and winter during which each bird was the least active (purple) at night. Note that the data for winter are plotted twice to facilitate comparison. In contrast to sleep restriction imposed by the experimenters (see Figure 6), sparrows maintained high levels of accuracy on the acquisition and performance tasks during periods of sleep restriction associated with migration. Furthermore, the total number of responses reached the highest values during the fall migration, in contrast to the decline in responding following experimenter-imposed sleep restriction.

Because the sparrows did not all migrate at exactly the same time, we also examined the effect of migratory status on accuracy and responding more specifically by selecting for each bird two 3-wk periods with maximal nocturnal activity during the spring and fall migrations, and two 3-wk periods with minimal nocturnal activity during the summer and winter nonmigratory periods (see Materials and Methods). We chose a 3-wk window for analysis, as this was the longest period of relative nocturnal quiescence that could be found in all birds during the summer. Accuracy on the repeated-acquisition task was virtually unaffected by migratory status. The total number of responses during the repeated-acquisition task was lowest during the winter, intermediate during spring and summer, and highest during the fall (Figure 8). The preservation of accuracy and responding during migration is thus unlike the decline in accuracy and responding observed following three nights of sleep restriction during the nonmigratory season.

## Discussion

Each spring and fall, songbirds switch from sleeping at night to migrating at night. Whether migrating songbirds sleep during flight, forgo sleep altogether, or compensate for night-time sleep loss by sleeping during the day has remained a mystery. Our EEG recordings of the white-crowned sparrow demonstrate a marked reduction in sleep, as well as distinct changes in sleep architecture during migration, including a shift in the timing of REM sleep to earlier in the night. Although an increase in drowsiness and a corresponding decrease in wakefulness were observed during the day, migrating sparrows did not compensate for sleep loss at night by sleeping more during the day or by increasing SWS intensity on migratory nights.

Despite the apparent reduction in sleep occurring during migration, observations of songbirds in the wild suggest that they are fully capable of maintaining a high level of cognitive and physical function, including navigation during long-distance flights, foraging, and evading predators in novel environments. Our results from the repeated-acquisition and performance tasks also suggest that songbirds are not cognitively or physically impaired during episodes of migratory restlessness in the laboratory. Unlike sleep restriction during the nonmigratory season, which caused a decrease in accuracy and responding in both the acquisition and performance components of the task, accuracy and responding did not decrease during migratory periods (spring and fall), when compared to nonmigratory periods (summer and winter). In fact, responding was highest during the fall migration and lowest during the winter.

We saw no evidence of sleep in active sparrows during periods of migratory restlessness in the laboratory setting, suggesting that songbirds do not sleep during migratory flights in the wild. Moreover, if birds have evolved the capacity to sleep while flying and depend upon it to avoid sleep deprivation, we would then expect them to be vulnerable to the effects of the sleep restriction resulting from migratory restlessness in the laboratory. The fact that birds exhibit migratory restlessness in captivity for periods of time similar to the duration of migration in the wild indicates an ability to withstand the effects of sleep restriction (Van Dongen et al. 2003). Furthermore, as discussed below, we did not see evidence of decrements in cognitive function similar to those observed following even a single night of sleep restriction during the nonmigratory season.

Despite the evidence against sleep in flight, our results do not rule out the possibility that some sleep might occur during flight in the wild. In the laboratory, migratory behavior is characterized by hopping and attempts to initiate flight, a time when sleep is not likely to occur. In the wild, however, it is conceivable that once birds have initiated a nocturnal flight, SWS could occur either unihemispherically or bihemispherically. Precedent for the former is found in bottlenose dolphins *(Tursiops truncates),* northern fur seals *(Callorhinus ursinus),* and cape fur seals *(Arctocephalus pussilus)* that swim in a coordinated manner while exhibiting unihemispheric SWS, a state characterized by SWS-related EEG in one hemisphere and EEG indistinguishable from wakefulness in the other hemisphere (Mukhametov et al. 1977; Lyamin and Chetyrbok 1992; Rattenborg et al. 2000). Birds also show interhemispheric asymmetries in SWS-related EEG when sedentary, although the asymmetry is less pronounced than that in aquatic mammals (Rattenborg et al. 2001). SWS may even occur simultaneously in both hemispheres during flight because the motor control of flight is mediated by spinal reflexes and can persist in decerebrated birds (Cohen and Karten 1974; Steeves et al. 1987); REM sleep during flight seems unlikely, however, given the associated reduction in skeletal muscle tone (Heller et al. 1983; Dewasmes et al. 1985). The fact that nocturnal flights occur high in the generally unobstructed night sky where constant visual assessment of the environment may not be necessary also makes SWS in flight seem feasible. In such a scenario, navigational assessments and corrections could be made during brief awakenings. Ultimately, recordings from birds migrating in the wild are needed to determine whether any sleep occurs during migratory flights.

The mechanisms that orchestrate the endogenous circannual rhythm of migratory behavior and associated changes in sleep remain largely unknown (Wingfield et al. 1990; Berthold 1996). Nevertheless, research into the neuroendocrine and circadian control of migratory behavior suggests possible interrelationships between migratory behavior and associated sleep patterns. Several studies suggest that migration is associated with increased hypothalamic–pituitary–adrenal (HPA) axis function (Meier and Fivizzani 1975; Ramenofsky et al. 1999; Wingfield 2003; Landys et al. 2004). In mammals, increased HPA function is associated with sleep disruption and REM sleep abnormalities. For example, plasma glucocorticoids are increased in rats (Meerlo et al. 2002) and humans (Spiegel et al. 1999) following sleep deprivation, and in patients with insomnia (Vgontzas et al. 2001). In depressed patients, increased plasma cortisol levels are correlated with reduced REM sleep latency (Poland et al. 1992). The reduction in sleep and shift in REM sleep timing observed during migration could therefore be related to activation of the HPA axis.

Changes in sleep may also be linked to alterations in the circadian rhythm during migration. In particular, since the occurrence of REM sleep is closely tied to the circadian rhythm in humans (Czeisler et al. 1980; Dijk and Czeisler 1995), the shift toward more REM sleep early in the night during migration may reflect a phase advance in the circadian propensity for REM sleep. In garden warblers *(Sylvia borin),* however, rather than being phase advanced, the amplitude of the circadian rhythm is reduced during migration (Gwinner et al. 1993). Nonetheless, a link between the timing of REM sleep and changes in the circadian rhythm may exist, since depressed humans with short REM sleep latencies show circadian patterns similar to migrating songbirds; the amplitude of the circadian temperature rhythm is reduced while the phase remains unchanged compared to subjects with normal REM sleep latencies (Schulz and Lund 1983). Gwinner suggested that a dampened melatonin rhythm allows the phase relationship of coupled activity rhythms to shift, thereby resulting in activity during the day and night (Gwinner 1996), a mechanism that may also contribute to the changes in total sleep time and REM sleep timing observed during migration.

Finally, the reduced latency to REM sleep during migratory nights might reflect a homeostatic response to prior REM sleep deprivation. In mammals, REM sleep deprivation or restriction leads to an increase in REM sleep during recovery sleep (Tobler 2000). As in mammals, pigeons *(Columba livia)* deprived of sleep also show an increase in REM sleep during recovery sleep (Tobler and Borbély 1988). Although the increase in REM sleep during the early portions of migratory nights is suggestive of a homeostatic response to prior REM sleep loss, the overall proportion of total sleep time spent in REM sleep was not consistently elevated on migratory nights. Consequently, the changes in REM sleep timing in migration are more suggestive of a shift in the circadian timing of REM sleep than a homeostatic response to REM sleep deprivation.

Regardless of the mechanism, the changes in sleep architecture during the night in migratory sparrows are reminiscent of those seen in individuals with mood disorders. Like migrating sparrows, both depressed and manic patients show reduced latency to REM sleep, loss of SWS, and reduced amounts of total sleep, often with early morning awakening (Benca et al. 1992); sleep decrements are most profound during mania. Given the aspects of bipolar illness, such as increased energy, activity, and creativity, that may be adaptive under certain circumstances (Andreasen 1987; Jamison 1993; Wilson 1998; Brody 2001), and its many parallels with migratory behavior, including seasonality, it is possible that similar mechanisms may be involved in both migration and bipolar disorder.

Despite the marked reduction in sleep during migration, we did not detect a significant increase in SWA during SWS on migratory nights, when compared to the corresponding hours of nonmigratory nights, in the five sparrows recorded during both nonmigratory and migratory states. This may indicate that migrating sparrows respond differently to sleep deprivation or that birds in general, unlike mammals, do not show increases in SWA during SWS following deprivation. The few studies that have examined sleep homeostasis in birds have produced conflicting results. Pigeons did not show a progressive decline in SWA (0.75- to 4.5-Hz) across the normal sleep period (SWS and REM sleep combined) when SWS was the predominant behavioral state, or an increase in SWA following 24 h of total sleep deprivation, suggesting a fundamental difference between sleep homeostasis in birds and mammals (Tobler and Borbély 1988). Unlike pigeons, however, blackbirds *(Turdus merula)* did show a decline in SWS-related SWA (0.5- to 4.0-Hz) across the major sleep period, indicating that aspects of mammalian SWS regulation may be present in some birds (Szymczak et al. 1996). In nonmigrating sparrows, we did not detect a decline in SWA during SWS across the night using the 0.75- to 4.5-Hz frequency range studied in pigeons and mammals, but a progressive decline in SWA was apparent in the 1.25- to 2.5-Hz frequency range, suggesting that this frequency band may reflect SWS homeostasis in sparrows. Nevertheless, even when this 1.25- to 2.5-Hz band was examined, migrating sparrows failed to show a consistent increase in spectral power.

Assuming that the decline in 1.25- to 2.5-Hz power in nonmigrating sparrows reflects SWS homeostasis, the apparent absence of an increase in this band during migration suggests that, unlike nonmigrating sparrows, migrating sparrows may require less SWS. A reduced need for SWS during migration may also be reflected in the shorter REM sleep latencies on migratory nights, since REM sleep latency is positively correlated with SWS need in humans (Feinberg et al. 1992). Alternatively, the increase in drowsiness occurring during the light phase in migrating sparrows may have compensated for SWS loss during the previous night, thereby accounting for the absence of an increase in SWS-related SWA during the subsequent night. Deprivation of daytime drowsiness may clarify whether this state contributes to SWS homeostasis in nonmigrating and migrating sparrows. In addition, a link between the declining trend in 1.25- to 2.5-Hz spectral power and SWS homeostasis in nonmigrating sparrows will need to be established with additional studies of SWS deprivation in both nonmigrating and migrating sparrows.

The results from the repeated-acquisition task suggest that songbirds appear resistant to the effects of sleep restriction during migration, although sleep restriction during the nonmigratory seasons appeared to impair accuracy and responding. It is possible that stress associated with the experimenter-induced sleep deprivation procedure may have contributed to the decrements observed; however, these birds were well acclimated to daily handling, and the methods used to deprive the birds of sleep (i.e., walking past the cage) were minimally intrusive. Regardless, birds in a migratory state were clearly able to maintain high levels of accuracy and responding during periods of spontaneous sleep loss occurring during migration. The only previous study to assess cognition in a migratory songbird compared the closely related nonmigratory Sardinian warbler *(Sylvia melanocephala momus)* to migratory garden warblers *(S. borin);* the migratory species performed better on a long-term memory task simulating habitat selection, despite being trained during a period of migratory restlessness, when sleep was presumably restricted (Mettke-Hofmann and Gwinner 2003). Although based only on a between-species comparison of one migratory and one nonmigratory species of songbird, these results and those from the white-crowned sparrow suggest that cognition is not impaired, and may even be enhanced, in migrating songbirds. Studies using other forms of neurobehavioral testing will be needed to determine whether migrating songbirds show a generalized resistance to the adverse effects of sleep restriction on specific cognitive functions known to be sensitive to sleep deprivation. In particular, the recent evidence suggesting that sleep is required for memory consolidation (Karni et al. 1994; Stickgold et al. 2000, 2001; Maquet 2001; Fischer et al. 2002; Fenn et al. 2003), a process not assessed with the repeated-acquisition task used herein, raises the question as to how birds consolidate memories during periods of migratory sleeplessness.

The apparent resistance to the effects of sleep restriction in migrating songbirds is unprecedented and clearly needs to be confirmed with further neurobehavioral testing. Future studies aimed at understanding the mechanisms underlying migratory sleeplessness may provide insight into the etiology and treatment of certain sleep disorders, as well as psychiatric disorders such as bipolar disorder, where similar seasonal bouts of sleeplessness with high levels of cognitive function are diagnostic of hypomania. Furthermore, an understanding of the mechanisms involved in migratory sleeplessness may lead to the development of methods to temporarily mitigate the effects of sleep deprivation that otherwise compromise performance in humans engaged in sustained operations where the maintenance of high levels of cognitive and physical function is critical. Finally, revealing the mechanisms through which migratory songbirds resist the effects of sleep deprivation may yield important clues as to the function of sleep in general.

## Materials and Methods

### 

#### Birds

Sparrows used for the operant testing were captured in Alaska (lat 64°49′ N, long 147°52′ E) during June 2002. Sparrows used for the EEG recordings were captured on their wintering grounds in the Sacramento valley in California (lat 39°00′ N, long 122°00′ E) during November 2002. All birds were collected using mist nets under the authority of a United States Fish and Wildlife Service permit. Birds were transported to the University of Wisconsin-Madison where they were individually housed in galvanized wire cages (L: 35 cm × W: 25 cm × H: 32 cm) in environmentally controlled rooms (L: 4.0 m × W: 2.7 m × H: 2.7 m; 22.0–24.5 °C, 40% relative humidity). Each bird was in visual and auditory contact with other birds in the room. To simulate seasonal changes in photoperiod, operant birds were exposed to photoperiods ranging from 9.5:14.5 LD to 16.5:7.5 LD. Sparrows used for the EEG recordings were maintained under a 12:12 LD schedule; lights went on at 06:00 with an illuminance level of 540–640 lux measured at the level of the cage floor. Illuminance during the dark phase was less than 0.5 lux. Birds were fed a mixed-seed and provided water ad libitum, and their diet was supplemented daily with lettuce, dried insects, live mealworms, and grit. Birds involved in operant testing were food restricted as described below. All experimental protocols were approved by the University of Wisconsin-Madison Animal Care and Use Committee.

#### Activity monitoring

As in previous studies (Wikelski et al. 1999), gross activity was measured using an infrared motion detector (no. 49–312, Radio Shack) connected to a system (VitalView version 4.0, Mini Mitter, Bend, Oregon, United States; http://www.minimitter.com) that logged the number of times that the bird crossed the infrared beam aimed across the center of the cage each 30-s interval. Although the infrared activity monitoring system may underestimate overall activity because it fails to quantify activity that does not result in a beam break, it nevertheless provides a rapid method for assessing gross seasonal changes in behavior. For the birds in which EEG was recorded, behavior was also continuously recorded using 16 infrared-sensitive cameras (two per bird) connected to a digital video storage system (Salient Systems, Austin, Texas, United States; http://www.salientsys.com). Infrared illuminators provided lighting for the cameras during the dark phase.

#### Surgery

In July 2003, eight adult white-crowned sparrows approximately 13–14 mo of age were randomly selected from our captive population and surgically instrumented for chronic EEG and EMG recordings. All surgical procedures were performed under isoflurane anesthesia (1.0%–3.5% isoflurane with 500 ml/min O_2_). The bird's head was stabilized in a Kopf Instruments (Tujunga, California, United States) stereotaxic device, using an adaptor developed for use with birds. A temperature-regulated heat pad set at 40 °C reduced heat loss during the procedure. After establishing a suitable anesthetic plane, the feathers overlying the cranium were clipped and the scalp was cleaned with 70% isopropyl alcohol. A longitudinal incision was made along the midline of the head to expose the cranium. After cleaning with 3% hydrogen peroxide and drying the cranium, four small holes were drilled through the cranium to the dura: two for the EEG electrodes, one for the reference electrode, and one for the ground electrode. To record the EEG from the left and right cerebral hemispheres, two holes were drilled 2 mm lateral of the midline, one over the left and one over the right Wulst, a brain region homologous to portions of the mammalian neocortex (Medina and Reiner 2000). A third hole for the reference electrode was positioned over the midline of the cerebellum. The fourth hole for the ground electrode was drilled over the right hemisphere. Stainless steel electrodes (no. AS 633, Cooner Wire, Chatsworth, California, United States) were inserted through the holes to the level of the dura and held in place using surgical glue. A final electrode was positioned over the nuchal muscles for recording EMG activity. Each electrode was connected to a lightweight, flexible, and electrically shielded recording cable (Dragonfly, Ridgeley, West Virginia, United States; http://www.dragonflyinc.com). The cable was attached to the bird's cranium using dental acrylic (Justi Products, Oxnard, California, United States). To form a strong adhesion, the acrylic was allowed to infiltrate the porous cavity between the inner and outer layers of the cranium through small holes drilled only through the dorsal layer of the cranium (Dave et al. 1999). Finally, the incision was closed around the acrylic with surgical adhesive (Tissuemend II, Veterinary Products Laboratories, Phoenix, Arizona, United States; http://www.vpl.com).

#### Electrophysiological recording

After surgery, each bird was placed in the recording cage (L: 35 cm × W: 25 cm × H: 32 cm) for at least 10 d of postoperative recovery and adaptation to the recording cable. The recording cable was attached to a low-torque, six-channel mercury commutator (Dragonfly) designed for use with small birds (Dave et al. 1999), and the weight of the recording cable was counterbalanced with a spring; these recording conditions allowed the sparrows to move unimpeded throughout the cage. The EEG and EMG signals were referenced to the cerebellar electrode, amplified, and band-pass filtered (0.3- to 30-Hz and 10- to 90-Hz, respectively), using Grass-Telefactor amplifiers (model 12 Neurodata and 7P511, Grass-Telefactor, West Warwick, Rhode Island, United States; http://www.grass-telefactor.com), digitized at 100 Hz, and visualized using Somnologica 3 software (Medcare, Reykjavik, Iceland; http://www.medcare.com).

#### Sleep–wakefulness scoring

Representative 24-h periods occurring prior to and during migration were selected for behavioral state scoring. The migratory nights selected for scoring were preceded by several nights with similar amounts of migratory restlessness. The behavioral state was scored visually, using both electrophysiological (i.e., EEG and EMG) and video recordings, and categorized as either wakefulness, drowsiness, SWS, or REM sleep. For accurate detection of REM sleep, the duration of scoring epochs was set at 4 s, since episodes of REM sleep may be as brief as only several seconds in birds (Rattenborg and Amlaner 2002). As in previous studies of sleep in birds (Rattenborg et al. 1999a, 1999b, 2001), the behavioral state was sampled across the 24-h period by scoring the first 4 s of each minute, resulting in a total of 1,440 samples per day. In addition, to determine the latency to SWS and REM sleep onsets precisely, every 4-s epoch was scored from lights out until the first unequivocal episodes of both SWS and REM sleep had occurred. Since SWS and REM sleep were restricted to the dark phase of the LD cycle in all birds during both migratory and nonmigratory seasons, sleep latency was calculated as the elapsed time from lights out to the first epoch of either SWS or REM sleep, and REM sleep latency was the elapsed time from the first epoch of SWS to the first epoch of REM sleep.

#### Spectral analysis of the EEG

EEG power spectra of the left and right hemisphere derivation were computed for all 4-s epochs, as described previously, by a FFT routine (Matlab function, Mathworks, Natick, Massachusetts, United States; using a Hanning window) within the frequency range of 0.25–25.0 Hz (Huber et al. 2000). Values were collapsed into 0.5-Hz bins. For the analysis of the time course of EEG power, artifact-free SWS epochs were selected.

#### Cognitive testing

To encourage responding by the sparrows, their food was restricted to maintain 90% of their free-feeding weights during nonmigratory periods. In practice, however, it was difficult to maintain birds at this weight, especially during periods of premigratory fattening, when even food-restricted birds gained weight. Sparrows were tested for 60-min sessions once per day on 5–7 d per week from 1 February 2003 through 15 February 2004. Testing was always performed between 09:30 and 16:00, and the testing order was counterbalanced across days for each bird. Activity levels in the home cages were measured using the infrared activity monitoring system. The amount of active time during the dark phase was used to select for analysis two 3-wk periods when the birds were migrating (spring and fall) and two 3-wk periods when they were not migrating (winter and summer).

#### Multiple schedule of repeated-acquisition and performance

Preliminary training for the repeated-acquisition task was described previously (Winsauer et al. 1995) and included shaping the approach to the food trough, shaping the response (key peck), and then reinforcing responses on each key when it was illuminated. To train repeated acquisition in all the sparrows, all three response keys were illuminated simultaneously with white light, but only one of the three response keys was chosen to be correct for a particular session, and each response emitted on that key resulted in the delivery of mixed-seed. Responding on either of the other two illuminated keys was considered an error and resulted in a 5-s time-out during which the key lights were extinguished and responding had no programmed consequence. For each daily session during this stage of training, the position for the correct response was varied pseudorandomly. After the sparrows acquired this task reliably, regardless of key position, a second response was added to the sequence or chain so that two correct responses were necessary to obtain seed. This type of sequential responding is procedurally defined as a “chain” because each response except the last produces a discriminative stimulus controlling the response that follows (Kelleher 1966). The key positions for the correct responses varied both within the two-response sequence and across sessions. The color of the key lights changed after each correct response. A third response was added to the sequence when stable responding was obtained under the two-response sequence. The average number of sessions required to train repeated acquisition of the first, second, and third member of the sequence was 38, 65, and 35, respectively. A second component was then added to the schedule so that sparrows responded under a multiple schedule of repeated acquisition and performance of response chains.

During acquisition components, the three response keys were illuminated at the same time with one of three colors: green, red, or white. Responding on the correct key in the presence of one color (e.g., keys green, center correct; keys red, left correct; keys white, right correct) changed the color of the key lights as well as the position for the next correct response. When the subject completed the response sequence by emitting three correct responses (i.e., one correct response in the presence of each color), the key lights were extinguished, and the stimulus light in the mixed-seed trough was illuminated for 0.05 s. Subsequently, the response keys were illuminated with the first color (i.e., green), and the sequence was reset. Within a given session, the correct response that was associated with a particular color did not change, and the same sequence (in this case, center-left-right [C-L-R]) was repeated during all acquisition components of a given session. Responding on this sequence was maintained by food presentation under a second-order FR3 schedule such that every third completion of the sequence resulted in the presentation of 5 s of access to mixed-seed. When sparrows responded on an incorrect key (in the example, the left or right key when the green lights were illuminated), the error was followed by a 5-s time-out. An incorrect response did not reset the three-response sequence (i.e., the stimuli and the position of the correct response were the same before and after a time-out).

To establish a steady state of repeated acquisition, the sequence was changed from session to session. An example of sequences for five consecutive sessions was C-L-R, L-R-C, C-R-L, R-L-C, and L-C-R. The sequences were carefully selected to be equivalent in several ways, and there were restrictions on their ordering across sessions. Briefly, each sequence was scheduled with equal frequency, and consecutive correct responses within a sequence were scheduled on different keys. Occasionally, a correct sequence position for a given color was the same for two consecutive sessions (in the list of sequences above, L-R-C and C-R-L).

During performance components, the response keys and the houselights were illuminated, and the sequence remained the same (R-C-L) from session to session. In all other aspects (color of the stimuli for each response in the sequence, second-order FR3 schedule of food presentation, 5-s time-out, etc.), the performance components were identical to the acquisition components. Experimental sessions always began with an acquisition component, which then alternated with a performance component after 20 reinforcers or 20 min, whichever occurred first. The performance component alternated back to the acquisition component after 10 reinforcers or 20 min, whichever occurred first. Each session terminated after 60 min.

#### Sleep deprivation

To determine whether accuracy and response rate on the repeated-acquisition and performance task were affected by sleep restriction during the nonmigratory season, sleep was restricted to the first 3 h of the dark phase (18:00–21:00) for three consecutive nights starting on 10 February 2004. Birds were deprived of sleep starting at 21:00 until the following day at 18:00 by experimenters who entered the housing room at least once every 5 min or sooner if behavioral signs of sleep were observed via closed-circuit cameras. Walking quietly past the cages was always sufficiently stimulating to keep the birds awake; we never had to handle the birds to induce wakefulness.

#### Statistics

Comparisons were made using either Student's *t*-test, with Welch correction for sample size, or ANOVA. All tests were performed using “R” (http://www.r-project.org). Prior to analysis two procedures were performed on the data for the cognitive testing. One consequence of counterbalancing the order of testing the birds was that the length of time from withdrawal of food to the onset of testing (and presumably one component of food motivation) varied on a 3-d schedule. For this reason a 3-d running average of the cognitive testing variables was computed. There was also a linear trend across the year, particularly in acquisition percentage correct (*r^2^* varied from 0.31 to as high as 0.67). This trend was removed before making seasonal comparisons.

The spring and fall migratory periods and the summer and winter nonmigratory periods were determined as follows. For each bird, each date during the study was used to compute the average nighttime activity for the following 21 d. The periods in the spring (21 February–20 May) and fall (21 August–20 November) with the highest average activity for each bird were designated as peak migration times, and the periods in the summer and winter with the lowest activity for each bird were chosen as nonmigratory times.

**Video 1:** Migratory Restlessness in a White-Crowned Sparrow Wing whirring while holding the perch (which occurred only at night) and perch hopping. Video from infrared camera. Bright object at the center of the screen is the source for the infrared motion detection beam.

**Video 2:** Drowsiness in a White-Crowned Sparrow A brief example of active wakefulness followed by about 50 sec of drowsy behavior. Captured during the daytime.

**Video 3:** SWS in a White-Crowned Sparrow Captured on surveillance camera. Bright object at the center of the screen is the source for the infrared motion detection beam.

**Video 4:** Acquisition Component of the Operant Task A correct sequence of three presses must be repeated three times. Feedback is provided by the lighted keys.

## Abbreviations

C: center
EEG: electroencephalographic activity
EMG: electromyographic activity
FFT: fast Fourier transform
HPA: hypothalamic–pituitary–adrenal
L: left
LD: light/dark
R: right
REM: rapid eye movement
SWA: slow-wave activity
SWS: slow-wave sleep
